# NASA’s Europa Clipper—a mission to a potentially habitable ocean world

**DOI:** 10.1038/s41467-020-15160-9

**Published:** 2020-03-11

**Authors:** Samuel M. Howell, Robert T. Pappalardo

**Affiliations:** NASA Jet Propulsion Laboratory, California Institute of Technology, Pasadena, CA USA

**Keywords:** Cryospheric science, Rings and moons

## Abstract

Jupiter’s satellite Europa almost certainly hides a global saltwater ocean beneath its icy surface. Chemistry at the ice surface and ocean-rock interface might provide the building blocks for life, and NASA’s Europa Clipper mission will assess Europa’s habitability.

One of the most powerful insights within planetary science in the last few decades is the discovery that celestial bodies throughout our solar system may hide vast global oceans of liquid water^1^. The first discovered likely ocean world beyond Earth, Europa, has captivated the science community and general public. With a launch date no later than 2025, NASA will return with the Europa Clipper mission to characterize Europa’s potential habitability by examining its ice shell and ocean, surface and atmospheric composition, geology and local-scale processes, and current activity.

The Voyager and Galileo missions first revealed a deformed surface at Europa with an average surface age younger than Earth’s^2^, dominated by water ice and renewed through recent or active geologic activity. The Galileo mission discovered Europa’s induced magnetic field, which indicates the presence of a global, electrically conductive fluid layer beneath the surface, most likely a saltwater ocean^3^. Recent observations also suggest the presence of plumes that may spew internal water into space^4–6^, indicating the potential for additional shallow liquid water reservoirs beneath Europa’s icy surface.

Geochemical constraints have led to open questions about the viability of Europa to host life. Intense radiation from Jupiter at Europa’s surface forms water and impurities into oxidants, chemical reagents capable of carrying out oxidation. Active geologic cycling of seawater through rocky material on the Europan seafloor is expected to be chemically reducing^7^. If mixing between the surface oxidants and the reduced ocean water occurs, there is an opportunity in Europa’s ocean or ice shell to produce a reduction–oxidation (redox) potential. All known life on Earth relies on such redox potentials to extract chemical energy from the environment in exchange for heat energy and entropy, enabling cellular maintenance, metabolism, and reproduction. Europa thus has the ingredients that may allow life to emerge: liquid water, bioessential elements, chemical energy, and a stable environment through time.

## Ice tectonics as a key to habitability

In order for Europa to maintain an ocean that is oxygenated like Earth’s, oxidants need to propagate downward from Europa’s icy surface^8,9^. One probable mechanism to achieve this is downward surface material transport through geologic processes. Hand et al.^8^ demonstrated that Europa could maintain an oxygenated ocean if downward cycling occurs only once every 30–70 Myr, and remain at least partially oxygenated with cycling every 500 Myr.

**Fig. 1:**
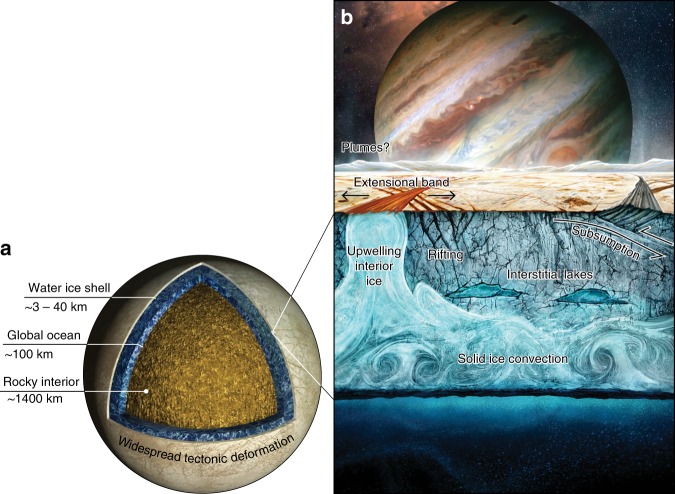
Cutaway views of Europa’s water ice shell and saltwater ocean. We show the scale of (**a**) the individual layers in relation to each other, and (**b**) an artistic schematic (artist: Jeff Nentrup) of different tectonic and geodynamic processes that might be at play within the ice shell today or in the past. Each of these processes may play a unique role in defining Europa’s habitability.

Whether or not Europa’s surface has been continually renewed throughout its history, or if some catastrophic process globally erased an ancient surface in relatively recent geologic times, are significant open questions that reflect our limited understanding of Europa’s icy shell. Currently, we understand that the shell is cold and stiff near the surface, producing fractures and faults in response to geologic stresses (Fig. 1). Where the ice shell is warm near the ocean interface, it may undergo solid-state ice convection (localized ice masses rising and falling buoyantly within the subsurface), a process akin to mantle convection on Earth.

Europa’s prominent bands are tabular extensional zones of ridges and grooves that transect the surrounding terrain, inferred to have opened through mechanisms analogous to mid-ocean ridge spreading on Earth^10^ (Fig. 1). Where the brittle ice is thick and strong, the ice rifts apart to form faulted bands that disrupt the original terrain^11^. Where the brittle ice is thin and weak, warm interior ice and incorporated ocean material may be exposed at the surface^11^.

In contrast to globally observed extensional processes, observations indicative of geologic mechanisms that remove old surface material are rare. A key discovery in this area was made by Kattenhorn and Prockter^12^, who found that an area of Europa’s surface ~100 km wide has been removed through an apparent subduction-like process. However, because subducted icy plates may quickly equilibrate with the shell’s interior^12^ (Fig. 1), Europa likely lacks the endogenic potential required to continuously renew its surface^13^.

Whether the surface is continually or discretely renewed, Europa’s tectonic processes are not the only modes of geologic transport acting on the body today. Another prominent and potentially active terrain on Europa, known as chaos, suggests the collapse of icy material to produce a mangled icy substrate on which fragments of the original surface have been translated. Leading hypotheses invoke convective transport of solid material^14^ or drainage of subsurface lakes^15^. While there is not yet consensus on the mechanisms of chaos formation, the terrain likely contributes to the downward transport of surface material.

Major difficulties remain in predicting the volume and frequency that material from the surface is conveyed to the interior ocean. If Europa’s surface was continually renewed by consistent endogenic processes, one would expect there to be an indication of repeated or cyclic geologic surface deformation. Instead, it appears that Europa has moved from global surface deformation, recorded in its ridged plains, to local formation of chaos and ridges^16^. This may indicate a globally perturbed body moving back toward equilibrium, expending the energy required for continued global deformation. With no definitive observations of near-surface melt and the rarity of overt convergent tectonics, future studies and observations of possible geologic transport mechanisms are critical to understanding Europa’s habitability.

## The Europa Clipper mission

**Fig. 2:**
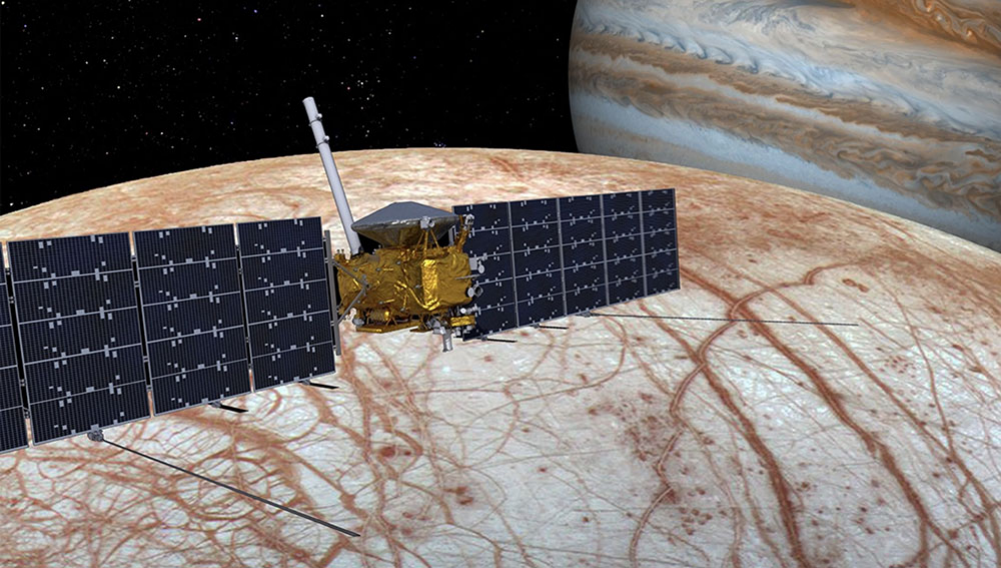
The Europa Clipper spacecraft. This computer-aided design view shows the present configuration of the spacecraft. Two arrays of solar panels extend outward from the spacecraft bus. Extending perpendicular to the solar arrays are the integrated ice-penetrating radar antennas. The silver dome is the primary communication antenna, and the boom extending upward from the spacecraft hosts the magnetometer sensors. The remaining scientific instruments are integrated onto the central structure.

The overarching goal of NASA’s Europa Clipper mission (Fig. 2) is to explore Europa to investigate its habitability. This goal traces to three mission objectives consisting of characterizing the ice shell and any subsurface water, including their heterogeneity, ocean properties, and the nature of surface–ice–ocean exchange; understanding the habitability of Europa’s ocean through composition and chemistry; understanding the formation of surface features, including sites of recent or current activity, and characterizing high science-interest localities.

In order to investigate Europa’s extremely thin atmosphere, Europa Clipper is equipped with an ultraviolet spectrograph, capable of detecting small plumes and providing valuable data about the composition and dynamics of the moon’s exosphere. Europa Clipper further carries a surface dust analyzer to measure the composition of small particles ejected from Europa, providing the opportunity to directly sample the surface and potential plumes on low-altitude flybys. The instrument is capable of identifying traces of ejected organic and inorganic compounds. Finally, the composition of Europa’s tenuous atmosphere and volatile surface materials ejected into space will be captured by a neutral gas mass spectrometer that can precisely determine composition.

For surface remote sensing, the Europa Clipper spacecraft is equipped with an imaging system consisting of a visible-spectrum wide- and narrow-angle camera suite that will map Europa. Europa Clipper also carries an imaging near-infrared spectrometer to probe surface compositions, relating it to the habitability of its ocean. Investigating Europa’s surface in mid- and far-infrared wavelengths, the thermal imaging system will provide high spatial resolution imaging to detect geologically active sites and potential plume vents.

Peering into the ice shell, Europa Clipper carries an ice-penetrating radar instrument that is designed to characterize and sound Europa’s ice shell from the near-surface to the ocean, revealing the three-dimensional icy structure and potential water pockets within. Ice shell thickness, ocean depth, and ocean salinity will additionally be evaluated by a conjunction of Europa Clipper’s plasma and magnetometer instruments. The plasma instrument measures the ionosphere surrounding Europa to characterize the magnetic fields generated by plasma currents, which can mask the magnetic induction response of Europa’s subsurface ocean. The magnetometer is a NASA-provided facility instrument with sensors that measure the direction and strength of magnetic fields in the spacecraft’s immediate vicinity. The interior of Europa will also be investigated via gravity and radio science, using Doppler data derived from the spacecraft’s telecommunication system.

## Outlook and future exploration

The Europa Clipper mission is currently in its implementation phase. The instrument and spacecraft designs are being finalized as the project approaches its Critical Design Review, and the first few pieces of the spacecraft bus are currently being manufactured. The integration of spacecraft components begins in 2021, and the spacecraft has a planned completion date of 2023.

In addition to the Europa Clipper mission, the European Space Agency’s JUICE mission will characterize the conditions that may have led to the emergence of habitable environments around Jupiter. While the primary focus of JUICE is Ganymede, the spacecraft will make two flybys of Europa, training complementary instruments at the icy moon. The JUICE mission will therefore be fundamental to understanding Europa within the context of the Jupiter system, and its comparative potential to host life.

Multiple mission concepts have arisen from the scientific community to take the next few steps in exploring Europa, including a spacecraft concept to touch its icy surface for the first time. The Europa Lander Science Definition Team proposed a science rationale focused on searching for biosignatures, supported by understanding Europa’s habitability, surface properties, and dynamics as the context^17^.

A mission concept for future decades would be to land a payload to provide direct access to the ocean below. Defining the technologies needed to accomplish this task has recently begun in NASA’s Scientific Exploration Subsurface Access Mechanism for Europa (SESAME) program, with focus on “cryobot” probes that could use a modified radioisotope or potential fission-power systems, ultimately exploring Europa’s ocean to attempt direct life detection.

The forthcoming decades promise fascinating discoveries at Europa and the other bodies believed to host alien oceans within our solar system. The Europa Clipper mission will take a profound step in advancing our understanding of habitable environments beyond Earth. Moving into the new decade, we are setting our sights forward to begin addressing the scientific and technological challenges uniquely associated with the robotic exploration of Europa and other icy ocean worlds.
